# hTERT promoter activity identifies osteosarcoma cells with increased EMT characteristics

**DOI:** 10.3892/ol.2013.1692

**Published:** 2013-11-19

**Authors:** LING YU, SHIQING LIU, WEICHUN GUO, CHUN ZHANG, BO ZHANG, HUICHAO YAN, ZHENG WU

**Affiliations:** 1 Department of Orthopedics, Renmin Hospital, Wuhan University, Wuhan, Hubei 430060, P.R. China; 2 Opening Laboratory for Oversea Scientists, Wuhan University School of Basic Medical Science, Wuhan, Hubei 430072, P.R. China; 3 Department of Radiation Oncology, Tumor Hospital Xiangya School of Medicine of Central South University, Changsha, Hunan 410013, P.R. China

**Keywords:** osteosarcoma, human telomerase reverse transcriptase, telomerase, epithelial-mesenchymal transition

## Abstract

Epithelial-mesenchymal transition (EMT) is a critical step in order for epithelial-derived malignancies to metastasize, however, its role in mesenchymal-derived tumors, i.e., osteosarcoma, remains unclear. Cancer stem cells (CSCs) are enriched with cells that undergo EMT. The activity of telomerase is maintained in normal stem cells and a number of malignant tumors. The current study observed the heterogeneity of telomerase activity among individual osteosarcoma cells. We hypothesized that telomerase-positive (TELpos) cells are enriched for stem cell-like and EMT properties. A human telomerase reverse transcriptase (hTERT) promoter-reporter was applied to assess the telomerase activity of individual MG63 osteosarcoma cells and sort them into TELpos and telomerase-negative (TELneg) subpopulations. It was found that the TELpos cells exhibited an enhanced ability to form sarcospheres *in vitro.* In addition, TELpos cells exhibited a higher expression of vimentin, accompanied by an increased long/short axis ratio. A panel of EMT-related genes was evaluated by quantitative PCR and western blot analysis, and were found to be significantly upregulated in TELpos cells. Next, the *in vitro* migration capacity was examined by Transwell assay, which confirmed that TELpos cells are more prone to migration (2.6 fold). The results of the present study support the concept that EMT also applies to mesenchymal-derived osteosarcoma and draws a connection between telomerase and EMT characteristics.

## Introduction

Osteosarcoma is the most common type of bone-forming malignant mesenchymal tumor, commonly present in the extremities of adolescents and young adults (1). Advances in chemotherapy and limb salvage surgery have improved the survival rates since the 1970s, however, drug resistance and lung metastases remain a great challenge for patients and clinicians (2).

Epithelial-mesenchymal transition (EMT) is a biological process in which epithelial cells lose their polarity and cell-cell adhesion, instead assuming a mesenchymal cell phenotype to gain increased migratory capacity and invasiveness. EMT is essential for implantation, embryogenesis and organ development, and is also involved in tissue regeneration and organ fibrosis (3). Recent studies have also shown that EMT is involved in cancer progression and metastasis, which allows the conversion of early-stage tumors into invasive malignancies. The loss of epithelial markers and the upregulation of mesenchymal genes confers the carcinoma cells with increased cell motility and invasive abilities (4–6).

Previous studies have demonstrated that EMT is tightly associated with the biology of cancer stem cells (CSCs). For example, CD44^+^CD24^−^/low cells are considered to be breast CSCs, which exhibit an EMT phenotype as characterized by the loss of E-cadherin expression and the gain of vimentin expression (7). TGF-β is a potential inducer of EMT, which causes the appearance of CD24^−^ cells from sorted pure CD24^+^ cell populations, accompanied by increased mesenchymal marker expression (8). However, the role of EMT in mesenchymal-derived sarcomas remains unclear.

Telomerase is a ribonucleoprotein that maintains integrity in the telomere regions, which shorten following each replication cycle (9,10). Telomerase is a reverse transcriptase enzyme composed of a catalytic component, human telomerase reverse transcriptase (hTERT) and telomerase RNA component (11). Telomerase activity is undetectable in the majority of human normal somatic cells. However, the activity of telomerase is maintained in stem/progenitor cells in self-renewal tissues (12). In addition, telomerase activation has been observed in ~90% of all human tumors, as well as in osteosarcoma, indicating that telomerase plays a key role in cancer development (13–15).

The significance of the role of telomerase in stem cells and cancer indicates that telomerase is more active in CSCs and cells that undergo EMT or vice versa. Cancer cells with stem-like and EMT properties exhibit higher telomerase activity. A recent study has shown that hTERT promotes cancer metastasis and recurrence by stimulating EMT and stemness of cancer cells (16).

The present study separated MG63 cells into two subgroups according to their telomerase activity and then investigated the EMT properties of each subgroup. The aim was to understand EMT in osteosarcoma and the correlation between EMT and telomerase.

## Materials and methods

### Chemicals and reagents

RPMI 1640, fetal bovine serum (FBS) and penicillin-streptomycin were obtained from Invitrogen Life Technologies (Carlsbad, CA, USA). The lentiviral transcriptional reporter vector, pGreenFire1-mCMV-EF1-Puro, was purchased from System Biosciences (Mountain View, CA, USA). The following antibodies for immunoblot and immunohistochemistry analysis were purchased from Santa Cruz Biotechnology, Inc. (Santa Cruz, CA, USA): Mouse monoclonal anti-E-cadherin, mouse monoclonal anti-pan-cytokeratin, mouse monoclonal anti-vimentin and mouse monoclonal anti-β-actin. The primary antibodies, rabbit polyclonal anti-N-cadherin, rabbit polyclonal anti-desmin, rabbit polyclonal anti-α-smooth muscle actin (SMA), mouse monoclonal anti-Twist1, rabbit polyclonal anti-Twist2 and rabbit polyclonal anti-Zeb2 were purchased from Abcam (Cambridge, MA, USA). The primary antibodies rabbit monoclonal anti-Snail, rabbit monoclonal anti-Slug and rabbit monoclonal anti-Zeb1 were purchased from Cell Signaling Technology, Inc.

### Cell culture

The human MG63 osteosarcoma cell line was purchased from the Shanghai Institute for Biological Sciences of the Chinese Academy of Sciences (Shanghai, China). The cells were cultured in RPMI 1640 supplemented with 10% (vol/vol) FBS and 1% (vol/vol) penicillin-streptomycin. The cells were propagated in a humidified environment at 37ºC with 5% CO_2_ and 100% humidity. Cell viability was determined using trypan blue stain (Invitrogen Life Technologies). For sarcosphere culture, the cells were plated in ultra-low attachment plates (Corning Inc., Corning, NY, USA) at a density of 5,000 cells/ml in RPMI 1640 supplemented with B27 supplement (Invitrogen Life Technologies), 10 ng/ml human epidermal growth factor (EGF; Sigma-Aldrich, St. Louis, MO, USA) and 10 ng/ml human basic fibroblast growth factor (bFGF; Sigma-Aldrich). Fresh aliquots of EGF and bFGF were added every other day. Following culture for 14 days, colonies containing >50 cells were regarded as sarcospheres and quantitated by inverted phase contrast microscopy.

### Lentiviral-reporter vector construction and infection

The hTERT promoter region (1.5 kb) (17–19) was cloned into the multiple cloning site of lentiviral vector, pGreenFire1-mCMV-EF1-Puro. HEK293 T cells were transfected with the constructs and then the viral supernatants were obtained. MG63 cells were infected with the viral supernatant and selected using puromycin (5 μg/ml) for one week to obtain stable reporter cells.

### Flow cytometry

The stable reporter MG63 cells were harvested using fresh 0.25% trypsin solution and resuspended in phosphate-buffered saline (PBS). The cells were maintained on ice prior to analysis. Green fluorescent protein (GFP) expression was assessed and the cells were sorted into telomerase-positive (TELpos) and telomerase-negative (TELneg) subpopulations according to their GFP expression status, using a Becton-Dickinson FACSort (San Jose, CA, USA).

### Telomeric repeat amplification protocol (TRAP)-ELISA

Telomerase activity was determined using the TeloTAGGG PCR ELISA PLUS kit (Roche Diagnostics GmbH, Mannheim, Germany), according to the manufacturer’s instructions. In brief, the cells were lysed and used for TRAP reaction. PCR products were then immobilized and detected. The absorbance of the samples was measured at 450 nm. Heat-treated cell extract was used as the negative control and a DNA template with the same sequence as a telomerase product with 8 telomeric repeats was used as a positive control.

### Immunocytofluorescence staining and microscopy

The sorted cells were seeded on square coverslips in six-well plates for 24 h to allow them to attach. Subsequently, the cells were fixed, permeated and blocked. The cells were then incubated with anti-vimentin antibody (diluted at 1:200) overnight at 4ºC. Phycoerythrin-labeled secondary antibody (Invitrogen Life Technologies) was applied for 1 h at room temperature. The cells were counterstained with DAPI and washed with PBS following each step of the staining procedure. Coverslips were mounted using ProLong Gold antifade reagent (Invitrogen Life Technologies). Images were captured using a Zeiss LSM 510 Meta confocal microscope (Carl Zeiss AG, Oberkochen, Germany). The long and short axes of cells were measured using the Zeiss LSM Image Examiner software (Carl Zeiss AG), and the long/short axis ratio was determined by counting 100 cells per experiment.

### Quantitative PCR

Total RNA was isolated and reverse transcribed. Quantitative (q)PCR was then performed using an ABI 7900 System (Applied Biosystems, Inc., Foster City, CA, USA) in the presence of SYBR Green. The primer sequences used for the PCR are listed in Table I. Target sequences were amplified at 95ºC for 10 min, followed by 40 cycles of 95ºC for 15 sec and 60ºC for 1 min. GAPDH was used as an endogenous normalization control. All assays were performed in triplicate. The fold change in mRNA expression was determined according to the 2^ΔΔCt^ method.

### Western blot analysis

Cell lysates were extracted using radioimmunoprecipitation assay lysis buffer containing protease inhibitor cocktail (Sigma-Aldrich). Protein concentrations were determined using the bicinchoninic acid method (Sigma-Aldrich). Cell lysates containing 40 μg protein were loaded and separated on 10% SDS-PAGE gels and subsequently transferred to polyvinylidene fluoride membranes (Invitrogen Life Technologies). The membranes were blocked and incubated at 4ºC overnight with primary antibodies diluted in 5% (w/v) skimmed milk powder in Tris-Buffered Saline with Tween 20 (Invitrogen Life Technologies). The membranes were then washed and incubated with secondary antibody at 1:5,000 dilutions for 1 h at room temperature. The membranes were again washed and developed using enhanced chemiluminescence substrate (Sigma-Aldrich).

### Wound healing assay

When adherent cells reached 80% confluence, a scratch was made using a 200-μl pipette tip. The cells were further incubated for 12 h and photomicrographs were captured under a phase contrast microscope (Nikon Eclipse TE2000-U; Nikon Instruments Co., Ltd., Shanghai, China).

### Transwell cell invasion assay

Assays were performed using a Matrigel-coated Transwell invasion assay plate (Corning Inc.). Assayed cells were placed in the upper chamber (1×10^5^ cells/well) in serum-free RPMI 1640. The lower chambers were filled with RPMI 1640 medium with 10% FBS. Upon the termination of the assay (24 h), the inserts were removed and the inner side was wiped with cotton swabs. The filters were stained with Harris’s hematoxylin solution (Sigma-Aldrich) and peeled off following washing and mounting the slides. The migrated cells were counted under a light microscope (Nikon Eclipse TE2000-U; Nikon Instruments Co., Ltd.).

### Statistical analyses

Each experiment was performed independently a minimum of three times. Data are presented as the mean ± SD. A two-tailed Student’s t-test was used to estimate intergroup differences if not otherwise stated. P<0.05 was considered to indicate a statistically significant difference.

## Results

### hTERT promoter reporter divides MG63 cells into two subpopulations

**Figure 1:**
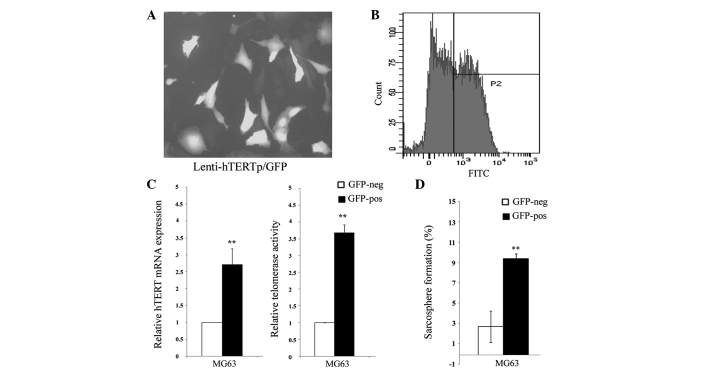
Osteosarcoma cells are heterogeneous according to their telomerase activity. (A) A representative observation of the fluorescence of MG63 cells following transduction. (B) The proportion of GFP-positive cells were analyzed using flow cytometry. (C) The MG63 cells were sorted according to their GFP status. hTERT mRNA levels were increased in the GFP-positive cells, and telomerase activity was also higher. (D) The sarcosphere formation capacity was significantly upregulated in the GFP-positive cells. ^*^P<0.01, vs. GFP-negative cells. hTERT, human telomerase reverse transcriptase; GFP, green fluorescent protein; FITC, fluorescein isothiocyanate.

The MG63 cells were transduced with the hTERT promoter reporter and selected for stably transduced cells. The expression of GFP was assessed by fluorescence microscopy and flow cytometry. The hTERT promoter was found to be heterogeneous among individual cells (Fig. 1A) and was sorted into two subpopulations by fluorescence-activated cell sorting, according to the hTERT promoter activity (Fig. 1B). It was further confirmed that these GFP-positive cells exhibit a significantly higher expression of hTERT and telomerase activity (Fig. 1C). Thereafter, the two different cell populations were named TELpos and TELneg, respectively. In addition, it was found that the sarcosphere formation capacity in the TELpos MG63 cells was greatly enhanced compared with the TELneg cells (Fig. 1D).

### TELpos cells are similar to mesenchymal-like cells

**Figure 2:**
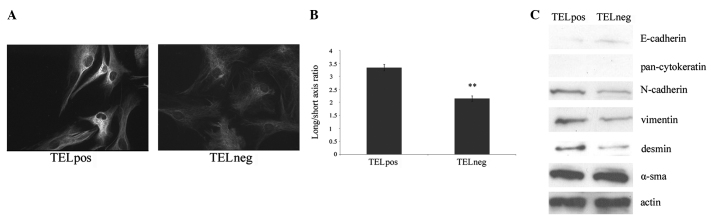
TELpos cells exhibit increased mesenchymal characteristics. (A) Representative immunofluorescence images of vimentin staining of TELpos and TELneg cells. (B) Long/short axis ratio of the two cell populations. (C) Western blot analysis of epithelial and mesenchymal markers of the two cell populations. TELpos, telomerase-positive; TELneg, telomerase-negative; SMA, smooth muscle actin.

The sorted TELpos and TELneg cells were stained using vimentin by immunocytochemistry. Vimentin expression was found to be higher in the TELpos cells compared with the TELneg cells (Fig. 2A). EMT is often accompanied by morphological alteration from a rounded phenotype to a spindle shape. Overall, the MG63 cells were spindle-shaped, however, round cells were also identified. Therefore, the long/short axis ratio was evaluated to assess the morphological differences. The TELpos cells were found to have an average ratio of 3.34, which is higher than that in the TELneg cells (average ratio, 2.14; Fig. 2B). In addition, the expression of common epithelial and mesenchymal markers was examined by western blot analysis. The epithelial marker, E-cadherin, was found to be increased in the TELneg cells, while cytokeratin was not detectable in the two groups. Mesenchymal-markers, including N-cadherin, vimentin and desmin, were upregulated in the TELpos cells compared with the TELneg cells. α-SMA was detected in the two groups, but no significant difference was found (Fig. 2C).

### TELpos cells exhibit increased expression of EMT driver genes

**Figure 3:**
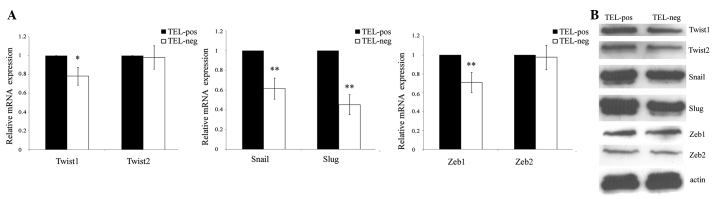
Analysis of EMT-driver genes. (A) qPCR analysis showing 4/6 genes were significantly upregulated in TELpos cells. (B) Western blot analysis results were consistent with the qPCR results. EMT, epithelial-mesenchymal transition; qPCR, quantitative PCR; TELpos, telomerase-positive; TELneg, telomerase-negative.

The EMT process is regulated by developmental transcriptional factors, which repress epithelial marker expression, but induce the expression of mesenchymal markers. The correlation between hTERT expression and these transcriptional factors, including Twist1/2, Snail, Slug and ZEB1/2, was investigated. In total, 4 out of 6 genes were found to be significantly upregulated in the TELpos cells by qPCR, in which Slug exhibited the highest average fold increase of 2.2. Twist2 and ZEB2 did not show any differences between the two cell groups (Fig. 3A). These results were confirmed by western blot analysis (Fig. 3B) and demonstrated that the high expression of hTERT is a predictor of an EMT-related gene signature.

### TELpos cells are more prone to migration and invasion

**Figure 4:**
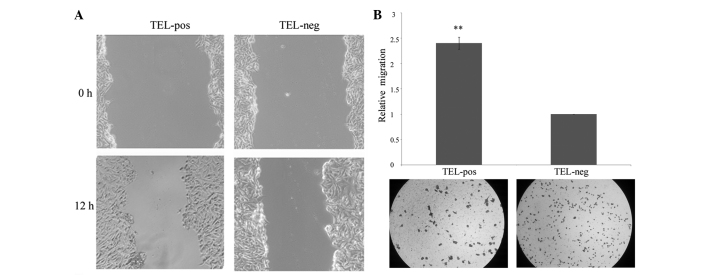
Analysis of migration and invasion capacity. (A) Wound healing assay showing that TELpos cells exhibited increased migration compared with TELneg cells after 12 h. (B) Transwell invasion assay showing that TELpos cells exhibited an increased capability to invade. TELpos, telomerase-positive; TELneg, telomerase-negative.

The difference in migratory and invasive capacity was investigated between the two cell populations. The TELpos cells exhibited significantly increased cell migration compared with the TELneg cells (Fig. 4A). The invasion potential through the Matrigel of the TELpos cells was also enhanced, with an average fold increase of 2.4 (Fig. 4B). This demonstrated that the high expression of hTERT correlates with increased cell motility.

## Discussion

Mesenchymal-to-epithelial transition (MET) is the reverse biological process to EMT, and has been shown to inhibit cancer progression. Silencing of the autocrine motility factor/phosphoglucose isomerase results in the MET of breast cancer, human lung fibrosarcoma and osteosarcoma cells, with reduced malignancy (20–22). However, whether the concept of EMT also applies to osteosarcoma remains unclear. The present study provided evidence that there is heterogeneity among osteosarcoma cells with regard to their EMT status, and demonstrated that hTERT promoter activity is a predictor for EMT characteristics.

The most commonly used assay for telomerase activity determination is the TRAP assay. hTERT is the core reverse transcriptase in telomerase holoenzyme, and the telomerase activity has been reported to correlate with the expression of hTERT. Therefore, hTERT expression levels are also used as an equivalent to cellular telomerase activity (23–26), however, these are not suitable for single cell analysis. Previous studies have shown that telomerase activity is not detectable in approximately two-thirds of osteosarcoma samples. Considering the evidence that heterogeneity widely exists in the tumor, we assume that in these TELneg samples, only a minority of the cells exhibited telomerase activity, which may lead to a false negative result. In addition, the previously described assays are not suitable for further functional confirmation. The present study constructed a lentiviral hTERT promoter-reporter to reflect the hTERT expression level and therefore, the telomerase activity. The results confirmed differential telomerase activity by determining the expression of hTERT and via the TRAP assay. Next, the MG63 cell population was divided into TELpos and TELneg subgroups for further experiments.

CSCs are considered to be involved in the initiation and progression of malignant tumors, and it has been confirmed by a number of previous studies that CSCs also exist in osteosarcoma. CSCs not only have the capacity of self-renewal and multipotent differentiation, but also contribute to metastasis and drug resistance (27,28). Considering the close connection between CSC and EMT, the present study tested whether different telomerase activity levels are associated with stem cell-like properties. TELpos MG63 cells were found to exhibit a significantly higher capacity to form sarcospheres *in vitro* compared with TELneg cells, which indicated an important role for hTERT in CSC self-renewal. Consistent with the results of the current study, it has been previously shown that CSCs exhibit abundant amounts of telomerase activity and that disruption of telomerase suppresses the self-renewal of CSCs (29). In addition, it has been found that, in gastric cancer, hTERT may regulate the activity of the canonical wnt pathway to modulate cancer stem-cell activity and EMT properties (16).

Experimentally, the activation of hTERT is a prerequisite for cellular immortalization and malignant transformation (12). In clinical studies, hTERT is expressed in 30% of primary osteosarcoma tumors and hTERT positivity is associated with tumor recurrence and decreased overall survival (13). The most well-defined mechanism for the requirement of telomerase is that tumor cells require telomerase to maintain telomere length. In addition, previous studies have shed light on the multiple biological functions during carcinogenesis independent of telomere-based activity. hTERT may induce the expression of vascular endothelial growth factor (30). Furthermore, overexpression of hTERT in normal stem cells may enhance their mobilization and proliferation, which is achieved by activation of the canonical wnt pathway (31). A novel hTERT function has been reported in gastric cancer, in which ectopically overexpressed hTERT promotes EMT and stemness, whereas knockdown by siRNA suppresses EMT and stemness (16). The observations of the current study also support the hypothesis that hTERT is involved in the process of the EMT of osteosarcoma. Firstly, TELpos cells exhibit a higher expression of mesenchymal markers, but a lower expression of epithelial markers compared with TELneg cells. Secondly, TELpos cells exhibit an increased long/short axis ratio, which indicates a mesenchymal phenotype. Thirdly, TELpos cells exhibit a higher expression of EMT-driver genes, including Snail, Slug, Twist and ZEB, compared with TELneg cells. Finally, TELpos cells are more likely to migrate and invade than TELneg cells. Collectively, the present study results revealed that osteosarcoma expresses a number of EMT-related genes, and these genes are heterogeneously expressed among individual cells. In addition, indirect evidence has been provided indicating that hTERT is involved in regulating the EMT program, which is different from its ability to maintain telomere length.

Consequently, therapies targeting telomerase may aid the elimination of EMT and CSCs, thereby preventing cancer progression. Future studies are required to evaluate the efficacy of telomerase inhibitors in the prevention of cancer recurrence, metastasis and drug resistance.

## Figures and Tables

**Table I:** Primer sequences used for qPCR.

Gene	Primer sequence
hTERT	
Forward	5′-GGAGCAAGTTGCAAAGCATTG-3′
Reverse	5′-TCCCACGACGTAGTCCATGTT-3′
Twist1	
Forward	5′-GCAGGACGTGTCCAGCTC-3′
Reverse	5′-CTGGCTCTTCCTCGCTGTT-3′
Twist2	
Forward	5′-GCAAGAAGTCGAGCGAAGAT-3′
Reverse	5′-GCTCTGCAGCTCCTCGAA-3′
Snail	
Forward	5′-GAGGCGGTGGCAGACTAG-3′
Reverse	5′-GACACATCGGTCAGACCAG-3′
Slug	
Forward	5′-CATGCCTGTCATACCACAAC-3′
Reverse	5′-GGTGTCAGATGGAGGAGGG-3′
Zeb1	
Forward	5′-CGAGTCAGATGCAGAAAATGAGCAA-3′
Reverse	5′-ACCCAGACTGCGTCACATGTCTT-3′
Zeb2	
Forward	5′-GGCGCAAACAAGCCAATCCCA-3′
Reverse	5′-TTCACTGGACCATCTACAGAGGCTT-3′
GAPDH	
Forward	5′-GAAGGCTGGGGCTCATTTG-3′
Reverse	5′-AGGGGCCATCCACAGTCTTC-3′

hTERT, human telomerase reverse transcriptase; qPCR, quantitative PCR.
